# Conditioned Medium from Bone Marrow Mesenchymal Stem Cells Restored Oxidative Stress-Related Impaired Osteogenic Differentiation

**DOI:** 10.3390/ijms222413458

**Published:** 2021-12-15

**Authors:** Ragda Saleem, Samih Mohamed-Ahmed, Rammah Elnour, Ellen Berggreen, Kamal Mustafa, Niyaz Al-Sharabi

**Affiliations:** 1 Center of Translational Oral Research (TOR)—Tissue Engineering Group, Department of Clinical Dentistry, Faculty of Medicine, University of Bergen, 5009 Bergen, Norway; Ragda.Saleem@student.uib.no (R.S.); Kamal.Mustafa@uib.no (K.M.); 2 Centre for International Health, University of Bergen, 5009 Bergen, Norway; 3 Department of Clinical Medicine, Faculty of Medicine, University of Bergen, 5009 Bergen, Norway; Rammah.Mustafa@uib.no; 4 Oral Health Centre of Expertise in Western Norway, 5009 Bergen, Norway; Ellen.Berggreen@uib.no; 5 Department of Biomedicine, Faculty of Medicine, University of Bergen, 5009 Bergen, Norway

**Keywords:** oxidative stress, mesenchymal stem cells, conditioned medium, antioxidant, osteogenic differentiation

## Abstract

Oxidative stress from high levels of intracellular reactive oxygen species (ROS) has been linked to various bone diseases. Previous studies indicate that mesenchymal stem cells (MSC) secrete bioactive factors (conditioned medium (MSC-CM)) that have antioxidant effects. However, the antioxidant role of MSC-CM on osteogenesis has not been fully studied. We aimed to identify antioxidant proteins in MSC-CM using mass spectrometry-based proteomics and to explore their effects on osteogenic differentiation of human bone marrow mesenchymal stem cells (hBMSC) exposed to oxidative stress induced by hydrogen peroxide (H_2_O_2_). Our analysis revealed that MSC-CM is comprised of antioxidant proteins that are involved in several biological processes, including negative regulation of apoptosis and positive regulation of cell proliferation. Then, hBMSC exposed to H_2_O_2_ were treated with MSC-CM, and the effects on their osteogenic differentiation were evaluated. MSC-CM restored H_2_O_2_-induced damage to hBMSC by increasing the antioxidant enzyme-SOD production and the mRNA expression level of the anti-apoptotic BCL-2. A decrease in ROS production and cellular apoptosis was also shown. MSC-CM also modulated mRNA expression levels of osteogenesis-related genes, runt-related transcription factor 2, collagen type I, bone morphogenic protein 2, and osteopontin. Furthermore, collagen type I protein secretion, alkaline phosphatase activity, and in vitro mineralization were increased. These results indicate that MSC-CM contains several proteins with antioxidant and anti-apoptotic properties that restored the impaired hBMSC osteogenic differentiation associated with oxidative stress.

## 1. Introduction

Bone is a complex, mineralized connective tissue made up of different types of cells that constantly interact together. Unlike other connective tissues, bone undergoes continuous remodeling through a balanced activity of osteoclasts and osteoblasts [1]. Many etrinsic insults (e.g., exposure to radiation or chemicals) and intrinsic insults (e.g., metabolic diseases) can lead to inflammatory response, poor blood flow, and ischemia and cause bone diseases related to loss of bone mass [2]. As critical mediators of pathophysiological responses, reactive oxygen species (ROS), including hydrogen peroxide (H_2_O_2_), superoxide, and hydroxyl radicals, have been considered as negative regulators of bone homeostasis via reducing osteoblast number and differentiation [3,4]. Based on these considerations, various antioxidants have been recommended to prevent the detrimental effects of ROS on osteogenic progenitors, thus promoting proper bone healing [5].

Certainly, the secret of the next revolution in the treatment of many diseases lies within each of us. Mesenchymal stem cells (MSC) are a group of cells of unique quality that can self-renew and have potential to differentiate into all bone cell types. MSC-based therapy can improve the healing of bone defects and even reverse clinically significant abnormalities in the skeleton [6,7,8]. However, current evidence indicates that ROS in the inflamed bone tissue may lead to impaired cell metabolism and decreased cell viability of engrafted MSC, which consequently limits their therapeutic effects and delays osteogenesis [9,10]. Therefore, protecting cells in vivo from apoptosis, together with enhancing their ability to survive under oxidative stress and promoting the endogenous healing process, is crucial.

Studies have ascribed the effect of MSC to a group of secreted bioactive molecules, including cytokines, chemokines, growth factors, and extracellular vehicles, collectively known as the MSC secretome. This secretome can be collected during in vitro culture of MSC, such as under serum-free culture conditions, and is defined as MSC-conditioned media (MSC-CM) with several biological properties, including anti-inflammatory, anti-fibrosis, antioxidant, and anti-apoptotic properties [11]. In previous studies, we have found that MSC-CM promoted the proliferation of endothelial cells in vitro and accelerated bone formation in distraction osteogenesis model in animals [12]. We have also shown that MSC-CM induced in vitro osteo/odontogenic differentiation of cultured dental pulp stem cells and positively modulated the healing process after tooth re-implantation in animals [13,14]. In particular, accumulating evidence indicates that bone ageing is associated with oxidative stress that is also known to be associated with osteonecrosis. In particular, MSC-CM have been shown to regulate local bone turnover on bisphosphonate-associated osteonecrosis of a jaw-like model [15]. Collectively, these studies indicate the effective role of MSC-CM in stimulating tissue regeneration and reducing healing time without causing inflammatory reactions. The therapeutic effect of MSC is mediated, at least partly, by secreted factors that stimulate migration of endogenous progenitor cells, promote cells differentiation, stimulate angiogenesis, and ultimately bone tissue repair and regeneration. However, this efficacy in regenerating bone under oxidative conditions still needs further investigation, as the capacity of bone-forming cells to achieve bone healing under such conditions is reduced.

To mimic the microenvironment of oxidative stress detected in various inflammatory bone diseases and to study the effect of oxidative stress on different cell types in vitro, H_2_O_2_ exposure is frequently used as a cellular model of oxidative stress [16,17,18]. Although, at low levels, H_2_O_2_ has a beneficial role in basic cellular activity such as proliferation and migration, high levels of H_2_O_2_ can cause oxidative stress and lead to adverse effects on cell activity [19,20]. Therefore, we hypothesized that MSC-CM could have an antioxidant property to protect cultured human bone marrow mesenchymal stem cells (hBMSC) against H_2_O_2_-induced cellular damage and impaired osteogenic differentiation. Therefore, the aims of this research were first to identify antioxidant proteins in MSC-CM and explore its effects on osteogenic differentiation of human bone marrow mesenchymal stem cells (hBMSC) exposed to oxidative stress induced by hydrogen peroxide (H_2_O_2_).

## 2. Results

### 2.1. Proteomics Revealed Expression of Antioxidant Proteins in MSC-CM

**Figure 1:**
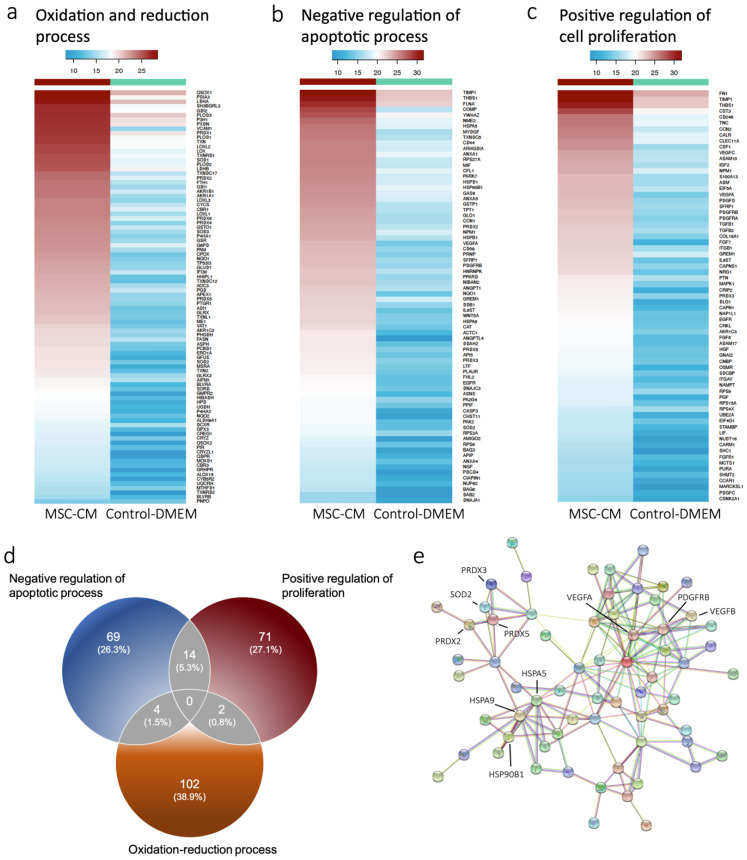
Proteins upregulated in MSC-CM were involved in oxidation, apoptosis, and proliferation processes. Heatmap of proteins that had involvement in enriched biological process according to gene ontology terms including (**a**) oxidation and reduction process, (**b**) negative regulation of apoptotic process, and (**c**) positive regulation of cell proliferation. Log2 protein abundance is shown. (**d**) Venn diagram depicting the number of proteins belonging to the different biological processes and overlap. (**e**) Network plot illustrating proteins involved in negative regulation of apoptotic process.

To profile the antioxidant proteins in our MSC-CM, mass spectrometry (MS)-based proteomics was performed. Overall, a total of 2218 proteins were identified by MS, and the data were subsequently investigated for differentially expressed proteins (DEPs) between MSC-CM and control-DMEM. Biological characterization of DEPs was then performed using the web-based tool DAVID for GO enrichment studies [21]. This analysis resulted in an enrichment of several biological processes including oxidation-reduction, negative regulation of apoptosis, and positive regulation of cell proliferation (Supplementary Table S1). Next, proteins involved in the identified GO terms were investigated for differential expression. Proteins included in the oxidation-reduction process were increased in MSC-CM (Figure 1a). Notably, this comprised several antioxidation enzymes including glutaredoxins (GLRX), peroxiredoxins (PRDX), and superoxide dismutases (SOD), out of which the latter two were also involved in the negative regulation of the apoptotic process (Figure 1b). Moreover, the platelet-derived growth factor (PDGFRB) and the vascular endothelial growth factor A (VEGFA) were included in both negative regulation of apoptotic process as well as positive regulation of cell proliferation process and found to be increased in MSC-CM (Figure 1b,c). Overall, the expression of proteins involved in these two processes was increased. Further, there was a clear overlap, as 14 proteins were involved in both terms (Figure 1d).

We then sought to explore protein-protein interactions in the data by using STRINGdb [22] and ranking-enriched GO terms according to strength. This analysis revealed an enrichment of peroxiredoxin, thioredoxin peroxidase activity, and platelet-derived growth factor binding terms (Supplementary Table S2), once again suggesting the involvement of growth factors and antioxidant enzyme function in MSC-CM. Furthermore, antioxidant enzymes (SOD2, PRDX2, PRDX3, and PRDX5) and growth factors (PDGFRB, VEGFA, and VEGFB) were both involved in negative regulation of apoptotic process and increased in MSC-CM, suggesting potential antiapoptotic effects. Similarly, the heat shock proteins HSPA9, HSPA5, and HSP90B1 were also involved in the regulation of apoptosis and increased in MSC-CM (Figure 1e).

**Figure 2:**
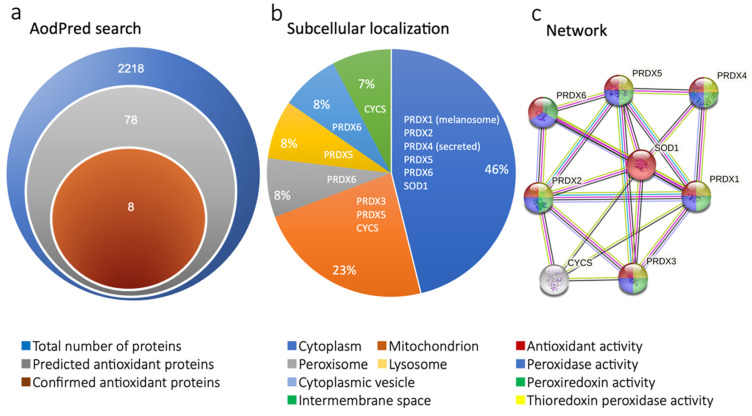

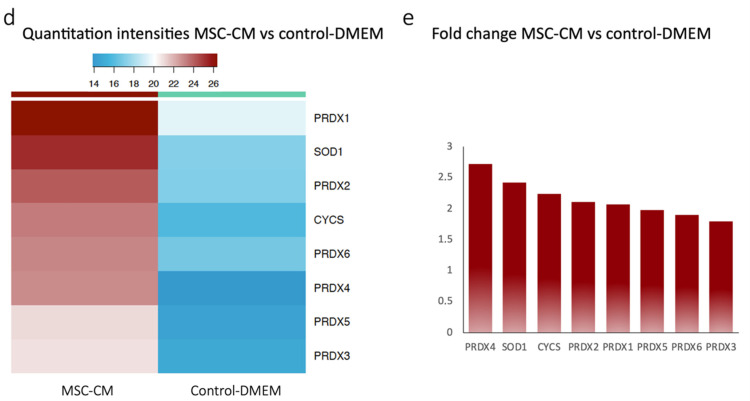
Identification and analysis of differential antioxidant protein expression using the Antioxidant Protein Database. (**a**) In total, 2218 proteins were identified by mass spectrometry, of which 78 were defined as predicted antioxidants and eight confirmed antioxidant proteins. (**b**) The subcellular localization of antioxidant proteins identified in the dataset as shown by a pie chart. (**c**) Network plot depicting protein-protein interactions between antioxidants and enrichment of molecular function terms in the dataset. (**d**) Antioxidant protein expression was increased when comparing MSC-CM to control-DMEM as shown by the heatmap of log2 raw protein abundance. (**e**) Bar plot of log10 fold change.

Importantly, the enrichment of oxidation GO terms was a common theme observed throughout the analysis of our data. Thus, further exploration of antioxidant protein expression was warranted. The Antioxidant Protein Database (AOD) was therefore used to further investigate the presence of antioxidant proteins [23]. Out of the 2218 proteins that were identified by MS, seventy-eight (78) proteins were categorized as predicted antioxidant proteins based on the AodPred query (Figure 2a). Eight (8) proteins were published in the AOD and therefore defined as confirmed antioxidant proteins and used for differential protein expression analysis (Supplementary Table S3). Unsurprisingly, the composition of identified antioxidant proteins was mainly represented by peroxiredoxins of which 6 were identified in this analysis as well as two other antioxidant proteins, cytochrome c (CYCS) and SOD. These findings were in line with the results from the enrichment analysis. Moreover, the subcellular localization of the detected antioxidant proteins was then shown by the AOD. The cytoplasm and mitochondrion were the main localization of the antioxidative proteins; however, localization in the peroxisome, lysosome, and intermembrane space was also shown (Figure 2b). STRINGdb was also used to depict protein-protein interactions between the proteins identified by AodPred (Figure 2c). The identified enzymes were all involved in antioxidant activity functions, as demonstrated in the figure. Further, all the enzymes apart from SOD were associated with peroxidase activity. Differential expression of AOD identified antioxidant proteins between MSC-CM and control-DMEM was then analyzed to further understand the mechanisms of MSC-CM effect. As demonstrated in the heatmap (Figure 2d) and barplot (Figure 2e), the overall observed pattern was an increase of antioxidant protein expression in MSC-CM, particularly PRDX4 and SOD1.

### 2.2. H_2_O_2_ Had Detrimental Effects on hBMSC

**Figure 3:**
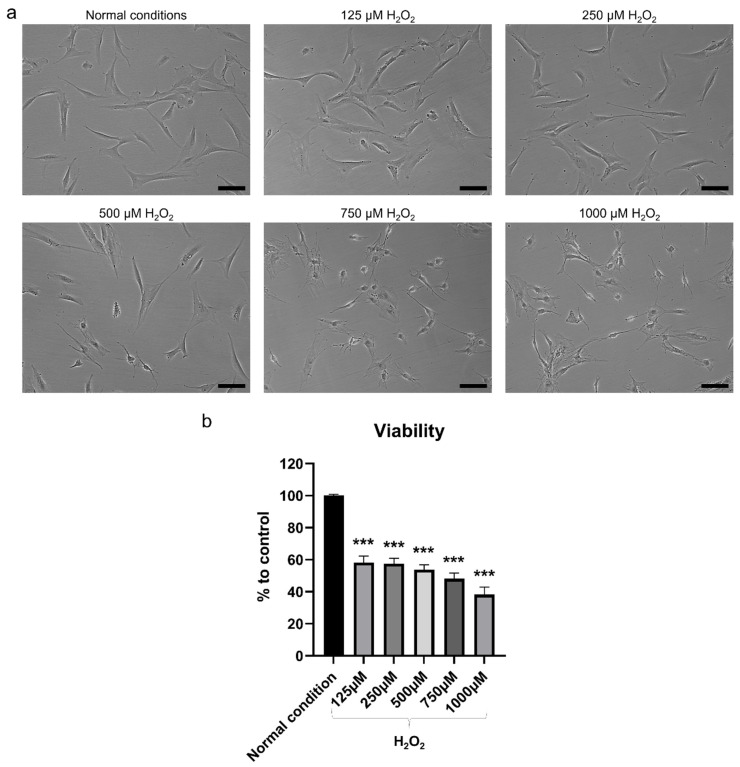
Effect of increasing concentrations of H_2_O_2_ on hBMSC viability. (**a**) Microscopic images showing changes in cell morphology in hBMSC after 2 h of exposure to increasing concentrations of H_2_O_2_. Scale bar 100 µm. (**b**) Viability of hBMSC evaluated by Alamar blue assay after 2 h of exposure to H_2_O_2_. *** *p* < 0.001.

**Figure 4:**
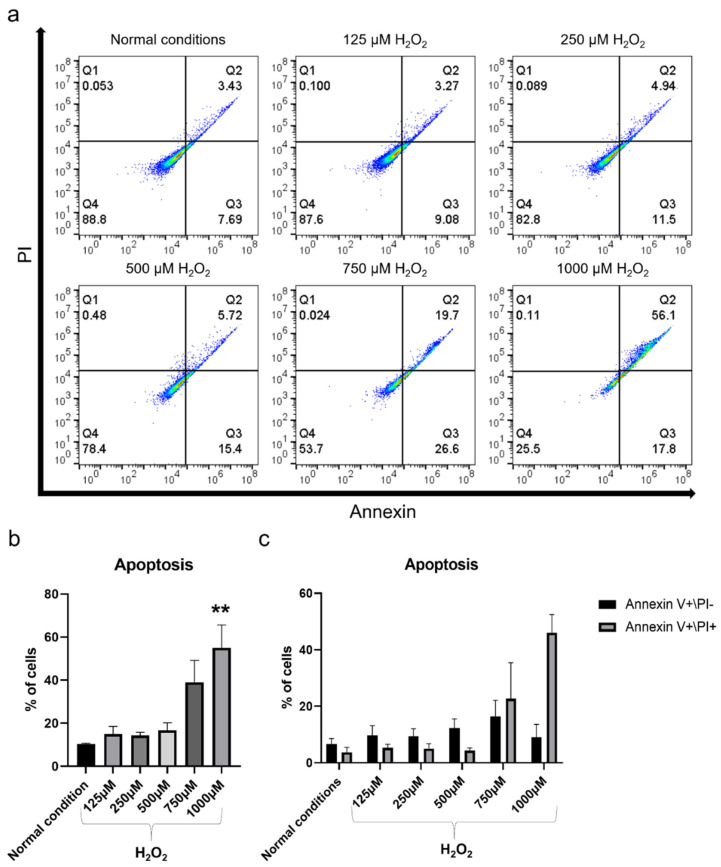
Effect of increasing concentrations of H_2_O_2_ on hBMSC apoptosis. (**a**) Detection of apoptotic cells by Annexin V/PI analysis using flow cytometry after 2 h of exposure to H_2_O_2_. (**b**) Percentage of apoptotic cells based on Annexin V/PI analysis after 2 h of exposure to increasing concentrations of H_2_O_2_. (**c**) Quantified data from Annexin V/PI analysis showing percentage of early apoptotic cells (Annexin V+/PI−) and late apoptotic cells (Annexin V+/PI+) after 2 h of exposure to H_2_O_2_. ** *p* < 0.01.

**Figure 5:**
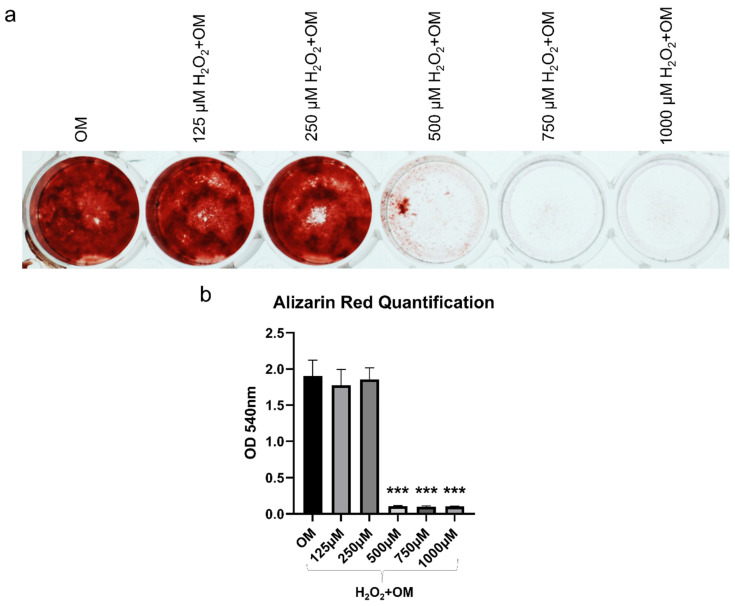
Effect of increasing concentrations of H_2_O_2_ on osteogenic differentiation of hBMSC. (**a**) Alizarin red staining after 21 days showing mineralization in hBMSC exposed to increasing concentrations of H_2_O_2_ for 2 h before adding OM. (**b**) Quantification of Alizarin red stain after 21 days in hBMSC exposed to H_2_O_2_ for 2 h prior to osteogenic differentiation. OM: osteogenic medium. *** *p* < 0.001.

After exposure to increasing concentrations of H_2_O_2_ for 2 h, the cellular changes in terms of morphology were proportional to the H_2_O_2_ concentration, with cells beginning to shrink and losing the spindle fibroblast-like morphology, and becoming more rounded (Figure 3a). The lower concentrations 125 and 250 µM H_2_O_2_ resulted in slight morphological changes, which became more pronounced at 500 µM H_2_O_2_. However, the morphological changes in the higher concentration were evident, indicating that most of the cells were dying. Exposure to H_2_O_2_ significantly reduced cell viability after 2 h of incubation (*p* < 0.001) (Figure 3b). The decrease in cell viability was also in direct proportion to H_2_O_2_ concentrations, with concentrations of 1000 and 750 μM H_2_O_2_ reducing the viability of hBMSC by more than 50% compared to non-exposed control cells. Notably, a concentration of 500 μM H_2_O_2_ caused a 46% decrease in cell viability, while lower concentrations of 250 and 125 μM H_2_O_2_ resulted in a 42% decrease in cell viability. The apoptotic effect of the H_2_O_2_ on hBMSC was observed based on Annexin V FITC/PI analysis. The percentage of apoptotic cells increased proportionally to the H_2_O_2_ concentration, reaching a significant level when 1000 μM H_2_O_2_ was used (*p* < 0.01) (Figure 4a–c). The percentages of early-stage apoptotic cells (Annexin V+/PI−) and late-stage apoptotic cells (Annexin V+/PI+) after exposure to 125 and 250 μM H_2_O_2_ were similar but showed a slight increase compared to the non-exposed control cells. A slight increase in the percentage of early-stage apoptotic cells was observed after exposure to 500 μM H_2_O_2_ in comparison to lower concentrations but not late-stage apoptotic cells. Exposure to 750 and 1000 μM H_2_O_2_ significantly increased the percentage of apoptotic cells compared to lower concentrations and this increase was mainly in late-stage apoptotic cells. For osteogenic differentiation, Alizarin Red staining showed poor mineralization in hBMSC after exposure to 1000, 750, and 500 μM H_2_O_2_ (Figure 5a,b). The amount of mineralization at these concentrations was significantly lower than in the OM-treated group (*p* < 0.001). Lower concentrations of H_2_O_2_ did not affect the osteogenic differentiation ability of hBMSC, as mineralization after exposure to 250 and 125 μM H_2_O_2_ was comparable to that of the OM-treated group. Therefore, exposure to 500 μM H_2_O_2_ was selected to test the therapeutic effect of MSC-CM afterwards, as this H_2_O_2_ concentration caused a 46% decrease in viability, moderate apoptotic effects, and a detrimental effect on osteoblast differentiation ability.

### 2.3. MSC-CM Reduced H_2_O_2_ Adverse Effects on hBMSC Viability and Apoptosis

**Figure 6:**
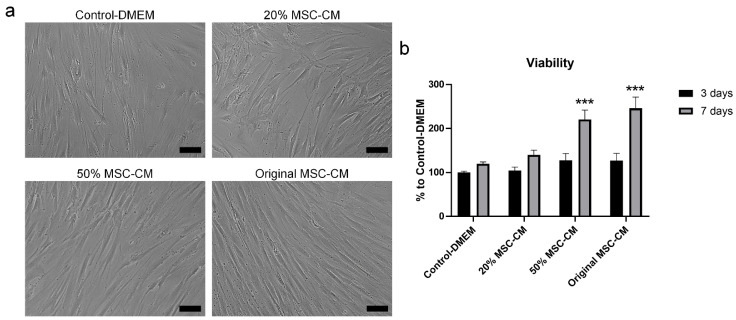
Effect of different concentrations of MSC-CM on viability of hBMSC exposed to H_2_O_2_. (**a**) Microscopic image showing cell morphology and growth of hBMSC exposed to H_2_O_2_ after 7 days of treatment with different concentrations of MSC-CM. Scale bar 100 µm. (**b**) Viability of hBMSC exposed to H_2_O_2_ after 3 and 7 days of treatment with MSC-CM using Alamar blue assay. MSC-CM: mesenchymal stem cells-conditioned medium; *** *p* < 0.001.

**Figure 7:**
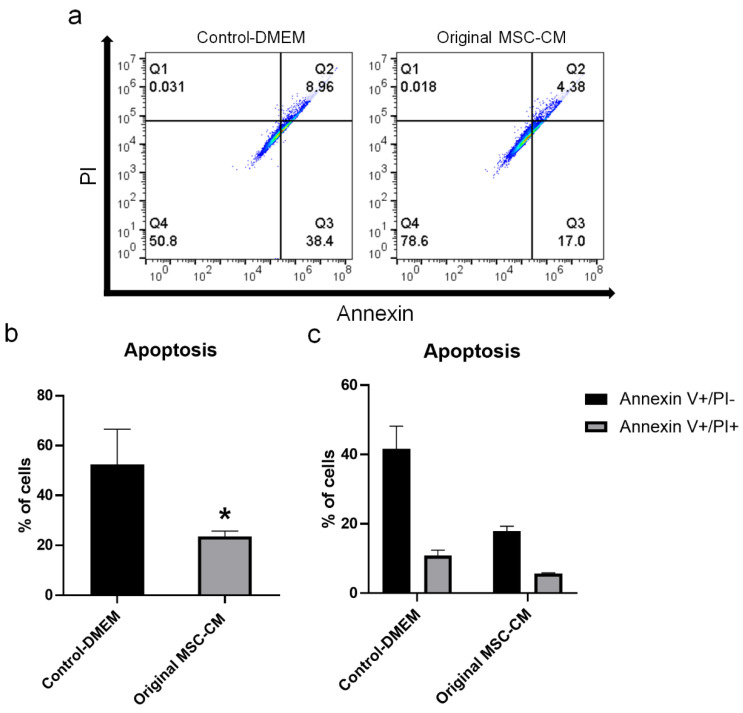
Anti-apoptotic effect of MSC-CM on hBMSC exposed to H_2_O_2_. (**a**) Detection of apoptotic cells in hBMSC exposed to H_2_O_2_ and treated with original MSC-CM and control-DMEM by Annexin V/PI analysis using flow cytometry for 7 days. (**b**) Percentage of apoptotic cells in hBMSC exposed to H_2_O_2_ and treated with original MSC-CM and control-DMEM for 7 days based on Annexin V/PI analysis. (**c**) Quantified data from Annexin V / PI analysis showing percentage of early apoptotic cells (Annexin V+/PI−) and late apoptotic cells (Annexin V+/PI+) in hBMSC exposed to H_2_O_2_ and treated with original MSC-CM and control-DMEM for 7 days. MSC-CM: mesenchymal stem cells-conditioned medium. * *p* < 0.05.

Cell viability increased after treatment of hBMSC exposed to H_2_O_2_ with 20%, 50%, and original MSC-CM for 3 days compared to control-DMEM, although not statistically significant (Figure 6a,b). An increase in cell viability from Day 3 to Day 7 was observed in all treated groups. However, this increase was more pronounced in the 50% and original MSC-CM-treated groups. After 7 days, treatment with 50% and original MSC-CM showed a statistically significant increase in cell viability of 100% and 125%, respectively, compared to the control-DMEM-treated group (*p* < 0.001). Although not statistically significant, a 20% increase in cell viability was observed under 20% MSC-CM treatment compared to control-DMEM. Based on this result, original MSC-CM were selected for use in further experiments. To test the effect of MSC-CM on rescuing the cells from H_2_O_2_ induced apoptosis, hBMSC exposed to H_2_O_2_ were treated with control-DMEM and original MSC-CM for 7 days. Treatment with original MSC-CM significantly reduced the percentage of apoptotic cells, compared to control-DMEM (*p* < 0.05) (Figure 7a–c). The percentages of early-stage apoptotic cells (Annexin V+/PI−) in the original MSC-CM-treated group were about 20%. This is significantly lower than in the control-DMEM-treated group, which had around 40% early-stage apoptotic cells. Similarly, the percentage of late-stage apoptotic cells (Annexin V+/PI+) in the original MSC-CM-treated group was 6%, which was less than 9% of the cells in the control-DMEM-treated group.

### 2.4. MSC-CM Exerted Therapeutic Effects by Increasing the mRNA Expression Level of the Anti-Apoptotic Gene BCL-2, Reducing ROS Generation, and Increasing the Production of the Antioxidant Enzyme-Sod

**Figure 8:**
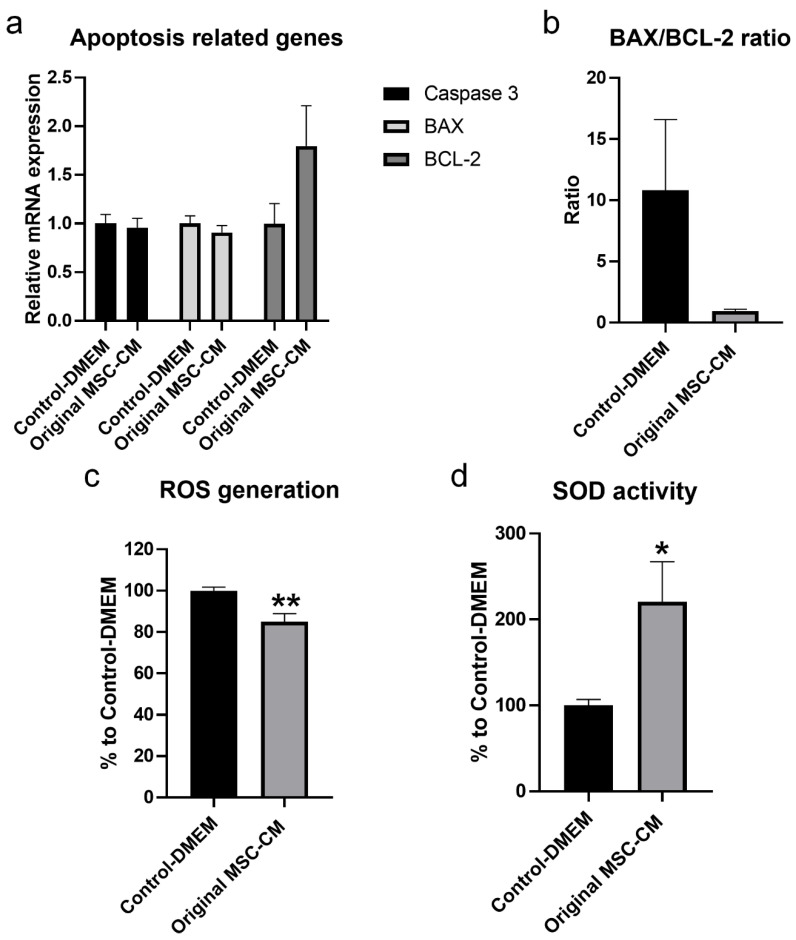
Effect of MSC-CM on mRNA expression levels of apoptosis-related genes, ROS generation, and production of the antioxidant enzyme-SOD on hBMSC exposed to H_2_O_2_. (**a**) mRNA expression levels of apoptosis-related genes caspase 3, BAX, and BCL-2 in hBMSC exposed to H_2_O_2_ after 3 days of treatment with MSC-CM and control-DMEM. (**b**) BAX/BCL-2 ratio of mRNA in hBMSC exposed to H_2_O_2_. (**c**) ROS generation in hBMSC exposed to H_2_O_2_ after 7 days of treatment with MSC-CM and control-DMEM. (**d**) Production of the antioxidant enzyme-SOD in hBMSC exposed to H_2_O_2_ after 7 days of treatment with MSC-CM and control-DMEM. MSC-CM: mesenchymal stem cells-conditioned medium; BCL-2: B-cell lymphoma-2; BAX: BCL-2-associated X protein; ROS: reactive oxygen species; SOD: superoxide dismutase. * *p* < 0.05, ** *p* < 0.01.

The mRNA expression level of the anti-apoptotic gene BCL-2 in hBMSC exposed to H_2_O_2_ treated with original MSC-CM for 3 days was significantly higher compared to control-DMEM (Figure 8a). A decrease in BAX/BCL-2 ratio to more than 1/10 in hBMSC exposed to H_2_O_2_ after treatment with MSC-CM was also detected (Figure 8b). As ROS plays a pivotal role in pro-apoptotic signaling cascades, the current study examined the effects of original MSC-CM compared to control-DMEM treatments on ROS generation at Day 7. Generation of intracellular ROS in the original MSC-CM-treated group was significantly lower than in the control-DMEM-treated group (*p* < 0.01) (Figure 8c). Generation of intracellular ROS in the original MSC-CM-treated group was decreased by 15% compared to the control-DMEM-treated group. Moreover, the production of the antioxidant enzyme-SOD was significantly higher in the original MSC-CM-treated group than in the control-DMEM-treated group (*p* < 0.05) (Figure 8d), where original MSC-CM treatment resulted in greater inhibition of cellular oxygen reduction by 100% compared to the control-DMEM-treated group (*p* < 0.05).

### 2.5. MSC-CM Restored the H_2_O_2_ Induced Inhibition of Osteogenic Differentiation of hBMSC

**Figure 9:**
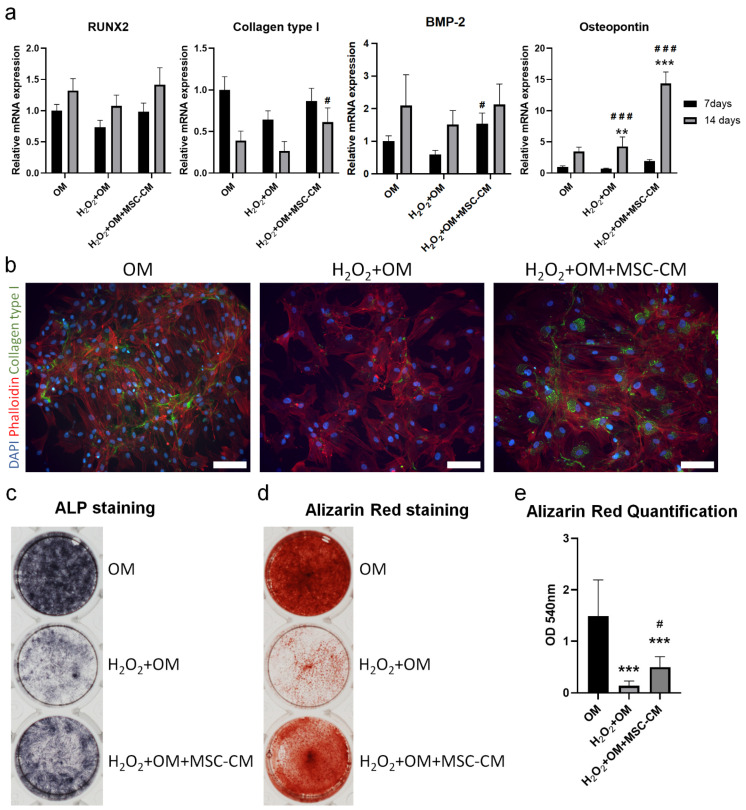
Effect of MSC-CM on osteogenic differentiation of hBMSC exposed to H_2_O_2_. (**a**) mRNA expression levels of osteogenic genes Runx2, Col 1α2, and SPP1 after 7 and 14 days. (**b**) Immunofluorescence staining of Col 1α2 after 7 days. (**c**) ALP staining after 14 days showing ALP activity. (**d**) Alizarin red staining after 21 days showing mineralization. (**e**) Quantification of Alizarin red stain after 21 days. Runx2: runt-related transcription factor 2; OM: osteogenic medium; MSC-CM: mesenchymal stem cells-conditioned medium. ALP: alkaline phosphatase. * Indicates a significant difference from OM, **#** indicates a significant difference from H_2_O_2_+OM, ** *p* < 0.01, *** *p* < 0.001, **#** *p* < 0.05, **###** *p* < 0.001.

To elucidate the effects of MSC-CM on the osteogenic differentiation capacity of hBMSC exposed to H_2_O_2_, in terms of mRNA expression levels of osteogenic-related genes, ALP activity, and mineralization, we cultured hBMSC in three different conditions: OM, H_2_O_2_+OM, and H_2_O_2_+OM+MSC-CM. We examined mRNA expression levels of osteogenesis-related genes runt-related transcription factor 2 (RUNX2), collagen type I (Col 1α2), bone morphogenic protein 2 (BMP-2), and osteopontin (SPP1) after 7 and 14 days (Figure 9a). After 7 days, the mRNA expression level of RUNX2 in the H_2_O_2_+OM-treated group was lower than in the H_2_O_2_+OM+MSC-CM-treated group, which was at a similar level to the OM-treated group. After 14 days, the mRNA expression level was increased compared to 7 days in all treated groups, but it followed the same trend and remained lower in H_2_O_2_+OM when compared to the H_2_O_2_+OM+MSC-CM- and OM-treated groups that had comparable expressions. The mRNA expression level of BMP-2 followed a similar trend to that of RUNX2. However, the H_2_O_2_+OM+MSC-CM-treated group had significantly higher expression than the H_2_O_2_+OM-treated group after 7 days. The mRNA expression level of Col 1α2 in the H_2_O_2_+OM+MSC-CM-treated group was moderately lower than in the OM-treated group, but higher than in the H_2_O_2_+OM-treated group after 7 days. After 14 days, the expression decreased in all-treated groups. However, the expression was significantly higher in H_2_O_2_+OM+MSC-CM than in H_2_O_2_+OM that had the lowest expression (*p* < 0.05) and was not significantly different from the OM-treated group. The mRNA expression level of SPP1 in the H_2_O_2_+OM+MSC-CM-treated group was significantly higher than in the OM-treated group (*p* < 0.01) and the H_2_O_2_+OM-treated group (*p* < 0.001) after 7 days. After 14 days, the expression was increased in all-treated groups, yet the H_2_O_2_+OM+MSC-CM-treated group had a significantly higher expression than the other treated groups (*p* < 0.001). Immunofluorescence staining at 7 days showed intracellular synthesis of Col 1α2 and secretion into the extracellular matrix (ECM) in the H_2_O_2_+OM+MSC-CM-treated group (Figure 9b). Although this secretion of Col 1α2 into ECM was less than that in the OM-treated group, it was substantially greater than in the H_2_O_2_+OM-treated group, which showed negligible secretion of Col 1α2. After 14 days, the highest ALP activity was observed in the OM-treated group (Figure 9c). However, considerably higher ALP activity was detected in the H_2_O_2_+OM+MSC-CM-treated group than in the H_2_O_2_+OM-treated group (Figure 9c). After 21 days, Alizarin Red staining revealed significantly greater mineralization in the OM-treated group than the other treated groups (*p* < 0.001) (Figure 9d,e). However, mineralization in the H_2_O_2_+OM+MSC-CM-treated group was significantly higher than the H_2_O_2_+OM-treated group (*p* < 0.05), which had limited mineralization.

## 3. Discussion

Previous studies have reported that MSC displayed remarkable therapeutic properties on bone tissue defects and diseases. Investigators have also proposed that paracrine factors secreted from MSC can mediate and regulate several events of osteogenesis, angiogenesis, cell migration, proliferation, and osteoblast differentiation [15]. However, the mechanisms of therapeutic antioxidant effect induced by MSC have not yet been well-defined. In this study, we have firstly demonstrated that human bone marrow MSC-CM possessed a group of signaling factors with antioxidant properties and reduced oxidative stress, and associated cellular apoptosis and subsequent impairment of osteogenic capacity of hBMSC.

Accumulation of ROS in cells, for example, due to ageing or traumatic tissue injury, leads to high intracellular concentration of ROS, which is greater than the capacity of intrinsic defense mechanisms in the cells, such antioxidant enzymes. This will lead to oxidative stress and cause cellular damage and induce apoptosis. H_2_O_2_ is an important ROS commonly used to study oxidative stress in vitro. In this study, we found a decrease in viability of hBMSC and an increase in cellular apoptosis after exposure to increasing concentrations of H_2_O_2_. This decrease in viability and increase in cellular apoptosis was associated with the increase in H_2_O_2_ concentration. However, when a concentration of 500 µm or higher was used, the toxic effect of H_2_O_2_ became clear, as hBMSC showed a substantial increase in cellular apoptosis and impaired osteogenic differentiation capacity. Although the viability of hBMSC was decreased after exposure to the lower concentrations of 125 and 250 µM, these two concentrations had a minimal effect on cellular apoptosis and osteogenic differentiation capacity of hBMSC. In line with our findings, previous study found that the effect of exposure to H_2_O_2_ on rat BMSC was also dose-dependent [16]. However, variation among studies in exposure time can contribute to variations in the results, as adverse cellular effects can be induced by lower concentrations of H_2_O_2_ when the exposure time is increased [17]. In addition, MSC from different sources may respond differently to H_2_O_2_, and this can be attributed to their capacity to adapt to oxidative stress in their original tissues [24].

Body tissues are susceptible to injuries throughout life. Accumulation of ROS, such as H_2_O_2_, in cells and tissues, leads to oxidative stress that can induce or exacerbate pre-existing damage and injury in the body tissues. The environment at the site of damage or injury involves cytotoxic events that inhibit cell function and can eventually lead to cell apoptosis. MSC role in the regeneration process is not only achieved through differentiation into cells of the injured tissues but also through paracrine signaling via their secretome that stimulates the local cells and modulates the environment to favor healing [25,26]. Our results showed that treatment of hBMSC exposed to H_2_O_2_ with different concentrations of MSC-CM restored the effect of H_2_O_2_ and increased the viability of these cells. This is comparable to results from previous studies showing a concentration dependent effect of MSC-CM in restoring the adverse effects of H_2_O_2_ on neural stem cells [27].

It is known that, under conditions of oxidative stress, endogenous antioxidants may not be sufficient to reduce or remove increased ROS. Therefore, several exogenous antioxidants have been investigated and are still being studied, including glutathione peroxidase (GPx) and/or dietary supplements containing polyphenols, among others [18,28]. In addition to other biological effects of MSC-secreting factors, MSC-CM have shown antioxidative effects in both in vitro and in vivo studies of different disease models [29]. MSC secrete various antioxidant enzymes such as SOD, catalase, and GPx, and factors including heme oxygenase-1 (HO-1) and glial-derived neurotrophic factor (GDNF) that lead to reduction of ROS and biomarkers of oxidative stress and enhancement of the antioxidant defenses [25,30]. Since identification of antioxidant proteins in MSC-CM is important, the current study confirmed the presence of several antioxidant proteins in MSC-CM, including PRDX1-6, SOD1, and CYCS. Similarly, Pires et al., reported that BMSC secretes PRDX1 and CYCS in addition to other antioxidant proteins [31]. Antioxidant proteins have various subcellular localizations, such as cytosolic and mitochondrial, and they can interact with each other to apply their antioxidant effects [28]. Our bioinformatics data revealed that the subcellular localization of the majority of the antioxidant proteins were the cytoplasm and mitochondria.

The secretome profile of MSC has been analyzed in several studies, and variations in the composition and concentration of secreted proteins might be attributed to the MSC source and their culture condition. A limitation of our study is that proteomics analysis was performed in MSC-CM pooled from six donors, although MSC were characterized based on stem cell characteristics suggested by ISCT. Studying the inter-individual variations in MSC-CM between donors as well as determining the number of donors to use for controlling such variations is important and needs further investigation. It has been reported that, under oxidative conditions, the use of MSC-CM increases cell viability and activity of antioxidant enzymes, while reducing ROS and apoptosis [27,29]. Likewise, the present study detected higher production of the antioxidant enzyme-SOD with decreased ROS generation in hBMSC exposed to H_2_O_2_ after treatment with MSC-CM. Although this current study did not show the mRNA expression level of the SOD gene after treatment with MSC-CM, we assumed that the high level of the antioxidant SOD activity after treatment with MSC-CM was produced from hBMSC exposed to H_2_O_2_, as confirmed by the low level of production of SOD after treatment with MSC-CM. It appears that the increased production of the antioxidant enzyme-SOD in hBMSC exposed to H_2_O_2_ was proportional to the decrease in ROS generation and was strongly involved in the antioxidant action of cells, thus increasing their survival rate. Indeed, the production and accumulation of free radicals in cells impairs their function and leads to permanent cellular damage and programmed cell death.

It is well-known that cell apoptosis process is controlled by the BCL-2 family of proteins that includes both anti-apoptotic members, namely BCL-2, and proapoptotic members, especially BAX, BCL-2 associated agonist of cell death (BAD), and BCL-2 homologous antagonist/killer (BAK) [32,33]. Activation of the proapoptotic factors BAX, BAD, and BAK activates other proapoptotic factors that subsequently activate the caspase family of proteins, especially caspase 3, which plays a crucial role in the execution of apoptosis [33]. On the other hand, BCL-2, an anti-apoptotic factor, plays an important role in the inhibition of the apoptosis process. After treatment with MSC-CM, a higher mRNA expression level of the anti-apoptotic gene BCL-2 than in the control-DMEM treated cells was detected. This was associated with slightly lower mRNA expression levels of pro-apoptotic genes, caspase 3 and BAX, in cells treated with MSC-CM. Generally, the ratio between the pro-apoptotic and anti-apoptotic factors, particularly BAX/BCL-2 ratio, inside a cell is known to be important for regulation of apoptosis and determines the fate of a cell in terms of death or survival [33]. We found a decrease in BAX/BCL-2 ratio to more than 1/10 in hBMSC exposed to H_2_O_2_ after treatment with MSC-CM. Similar findings have been reported by other studies [34]. For example, Xu et al. found lower mRNA expression level of the proapoptotic gene BAX and higher mRNA expression level of the anti-apoptotic gene BCL-2 in H_2_O_2_ exposed cells after treatment with umbilical cord MSC-CM compared to the untreated control cell [34]. In our proteomic analysis of MSC-CM, several other proteins with various biological effects were detected, including negative regulation of apoptotic process and positive regulation of cell proliferation process. Moreover, the bioinformatic analysis demonstrated that the growth factors detected in the MSC-CM, such as PDGFRB, VEGFA, and VEGFB, together with the antioxidant enzymes (PRDX and SOD), can act on inhibiting the apoptotic pathway and enhancing the survival of the cells [35,36]. It has been shown previously that CM from human MSC had a significant promoting effect on survival and neuronal density in retinal explants, which was attributed to the enrichment of factors of the PDGFR family, compared to human fibroblast-CM [35]. We believe that the inhibition of apoptosis by MSC-CM may also be related to the presence of heat shock proteins (HSPs) in MSC-CM. HSPs are known for their cytoprotective activity in response to cellular stress and ROS. It has been reported that HSPs work together with antioxidant systems in order to inhibit or nullify the effects of ROS and by extension impair apoptotic mechanisms [37]. Therefore, we assumed that the presence of confirmed and predicated antioxidant proteins and other biological factors with anti-apoptotic effects in MSC-CM may synergistically protect cultured hBMSC from H_2_O_2_-induced oxidative injury. However, the mechanism underlining the antioxidant and anti-apoptotic network effects of MSC-CM needs further investigation.

Oxidative stress has been linked to the ageing process of the body and many bone diseases, such as osteoporosis, joint inflammatory diseases, bone tumors, and complicated healing of bone fracture [4]. For instance, a strong relation between oxidative stress and osteoporosis has been found, since decreased bone mineral density appears to be associated with increased total ROS and decreased antioxidants in plasma [38]. It has been documented that high levels of ROS disturb the balanced osteoclasts and osteoblasts activity, which is responsible for the bone remodeling process, favoring bone resorption, and eventually leading to osteoporosis [3]. This is achieved not only by promoting osteoclast differentiation and activity [39] but also by inhibiting osteoblast differentiation [40]. Although several studies have discussed the therapeutic effects of CM and/or its derivatives from various sources of stem cells [41,42] in oxidative stress-induced bone tissue damage, little is known about the therapeutic effect of MSC-CM against H_2_O_2_-induced oxidative injury and impaired osteoblast differentiation capacity of cultured MSC. Therefore, in our study, we wanted to test the effect of MSC-CM on the impaired osteogenic differentiation capacity of hBMSC due to oxidative stress in vitro. We tested the mRNA expression level of four genes, RUNX2, Col 1α2, BMP-2, and SPP1, which are important in the osteoblastic differentiation that involves three phases, proliferation, matrix maturation, and mineralization [43], and found a higher mRNA expression level of these genes in hBMSC exposed to H_2_O_2_ treated with MSC-CM than in the control-DMEM. The higher mRNA expression level of Col 1α2 in hBMSC exposed to H_2_O_2_ after treatment with MSC-CM was confirmed at the protein level by findings from immunofluorescence staining, with both intracellular and extracellular secretion. Moreover, when ALP activity was evaluated, higher ALP activity was detected in hBMSC exposed to H_2_O_2_ after treatment with MSC-CM than in the control-DMEM treated cells. Subsequently, greater mineralization was formed in hBMSC exposed to H_2_O_2_ after treatment with MSC-CM than in the control-DMEM. Our results indicate that MSC-CM have the capacity to restore the diminished osteogenic capacity of hBMSC due to oxidative stress. Other studies also found profound effects of MSC-CM in recovering osteogenic capacity in oxidative stress-related bone diseases [15,44,45]. Oxidative stress also can have a role in the development of other bone diseases, such as osteonecrosis [46]. Therefore, Ogata et al., tested the effect of MSC-CM in osteonecrosis both in vitro and in vivo using an osteonecrosis model in rat jaw [15]. They found that application of human-derived MSC-CM had a protective effect on rat MSC and enhanced the mRNA expression level of osteogenesis-related genes in vitro and resulted in complete bone healing with soft tissue coverage in vivo. They attributed the effect of MSC-CM in achieving healing in their model to the antiapoptotic and anti-inflammatory properties of MSC-CM. In addition to the antioxidant, antiapoptotic, and anti-inflammatory factors, MSC-CM contains numerous cytokines and growth factors that play a pivotal role in the osteogenesis process [42]. Moreover, MSC-CM contains potent angiogenic factors, such as VEGF, that play a prominent role in angiogenesis, which is vital for the bone regeneration process [36,47].

## 4. Materials and Methods

### 4.1. Expansion and Characterization of hBMSC

Previously isolated hBMSC under ethical approval from the Regional Committee for Medical and Health Research Ethics in Norway (2013/1248/REK sør-øst C) were expanded in growth medium (Dulbecco’s Modified Eagle’s medium (DMEM, Thermo Fisher Scientific, Bleiswijk, Netherlands) supplemented with 10% fetal bovine serum (FBS, Sigma-Aldrich, St. Louis, MO, USA) and 1% antibiotics, penicillin/streptomycin (Sigma-Aldrich, St. Louis, MO, USA) and maintained at 37 °C in a humidified condition containing 5% CO_2_. The growth medium was changed twice a week. When 70–80% confluent, cells were detached using Trypsin/EDTA solution (Lonza, Verviers, Belgium) and then sub-cultured. Cell number and viability were assessed using 0.4% Trypan blue stain (Thermo Fisher Scientific, Eugene, OR, USA) and Countess 3 cell counter (Thermo Fisher Scientific, Singapore), and their morphology was observed using an inverted microscope (Eclipse TS100, Nikon, Tokyo, Japan). hBMSC were used at passages 3–5 for different experiments. hBMSC were characterized based on immunophenotype and multi-differentiation capacity according to the minimal criteria proposed by the International Society for Cellular Therapy (ISCT) to define human MSC (Supplementary Figure S1a–c) [48,49]. In total, human BMSC from nine donors were used in this study (six donors for preparation of MSC-CM and three donors for in vitro experiments).

### 4.2. Preparation of MSC-CM

To obtain MSC-CM, hBMSC at passages 3–5 were seeded at a seeding density of 5 × 103 cells/cm^2^ and cultured in a growth medium until 70–80% confluent. After three washes with PBS (Thermo Fisher Scientific, Bleiswijk, Netherlands), cells were cultured in serum-free DMEM for 48 h. Then, the medium was collected and centrifuged at 300× *g* for 5 min, and the supernatant was collected and re-centrifuged at 2000× *g* for 20 min to remove cell remnants and apoptotic bodies. The supernatant was filtered through a 0.2 μm pore size filter and identified as original MSC-CM. The MSC-CM from six donors were pooled, aliquoted, and stored at −80 °C. Control media defined as control-DMEM were produced by collecting serum-free DMEM incubated for 48 h without cells and underwent a similar process to the original MSC-CM.

### 4.3. Liquid Chromatography with Tandem Mass Spectrometry (LC-MS/MS) Analysis

To identify antioxidant proteins derived from the pooled MSC-CM compared to the control-DMEM, 10 mL of each were analyzed using LC-MS/MS. Briefly, CM were concentrated by ultrafiltration using Amicon Ultra-15 3kDa cutoff centrifugal filters (Sigma-Aldrich, St. Louis, MO, USA) prior to proteomic sample preparation. The protein concentration was measured using Qubit Protein Assay (Thermo Fisher Scientific, Waltham, MA, USA), and 10 μg protein was processed to obtain tryptic peptides, as described in Elise Aasebø et al. [50]. About 0.5 μg protein as tryptic peptides was analyzed by a linear quadrupole ion trap-orbitrap (LTQ-Orbitrap Elite) mass spectrometer (MS; Thermo Scientific, Bremen, Germany) equipped with a nanospray Flex ion source (Thermo Scientific, Sunnyvale, CA, USA), as previously described [50].

### 4.4. Bioinformatic Analysis and the Antioxidant Protein Database

Mass spectrometry (MS)-based proteomics was used to investigate differential protein expression between MSC-CM and control-DMEM. Biological characterization of the data was conducted by using the Database for Annotation, Visualization, and Integrated Discovery Database (DAVID) to investigate enrichment of gene ontology terms, biological processes, molecular function, and cellular component. STRING software [23] was used to explore protein-protein interactions in the dataset. Four protein-protein interaction analysis methods were used in STRINGdb: textmining, experimental, database, and co-expression [22]. A minimum interaction score of 0.7 was applied. False discovery rate (FDR)-corrected *p*-values were determined using the Benjamini–Hochberg procedure. The MS data were then investigated for presence of antioxidant proteins by using the Antioxidant Protein Database (AOD). Briefly, protein IDs were converted to FASTA format sequences and used as input for AodPred search. AodPred is a support vector machine-based method, used to query and predict possible antioxidant proteins [21]. The antioxidant status of the predicted proteins was then validated by confirming their record in the published AOD database. AOD was also used to detail subcellular location and function of confirmed antioxidant proteins. Antioxidant proteins identified in AOD were then further analyzed for differentially expressed proteins (DEPs). Visualization of the search results and data analysis was performed in R (version 3.2.2). Quantitative proteomic expression intensities were demonstrated by generating heatmaps using the heatmap.2 function provided in the gplots package (version 3.1.1) in R.

### 4.5. Exposure to H_2_O_2_ and MSC-CM Treatments

To establish the oxidative stress model, hBMSC were exposed to increasing concentrations of H_2_O_2_ (Sigma-Aldrich, St. Louis, MO, USA) (125, 250, 500, 750, and 1000 µM) in DMEM with 1% FBS at 37 °C with 5% CO2 for 2 h. To investigate the therapeutic effects of MSC-CM after H_2_O_2_ exposure, cells were washed with PBS and incubated with different concentrations of MSC-CM (20%, 50%, and original MSC-CM) for 3 and 7 days. Control-DMEM was used as a control-treated medium. To ensure adequate nutrients were available, media containing 1% FBS were changed every 24 h.

### 4.6. Cell Viability Assay

Cell viability after exposure to increasing concentrations of H_2_O_2_ for 2 h and after exposure to 500 µM H_2_O_2_ for 2 h followed by treatment with control-DMEM, 20%, 50%, and original MSC-CM for 7 days was examined using the Alamar blue cell viability assay (Thermo Fisher Scientific, Eugene, OR, USA) according to the manufacturer’s instructions. After incubation for 2 h at 37 °C with 5% CO_2_, the reduction of Alamar blue solution was measured by fluorescent at 560 nm excitation and 590nm emission using Varioskan LUX Multimode Microplate Reader (Thermo Fisher Scientific, Vantaa, Finland). The cell viability assay was assessed according to the following formula: viability (%) = OD of experimental group/OD of control-treated group × 100.

### 4.7. Flow Cytometry Analysis of Cell Apoptosis

The apoptotic effect of increasing concentrations of H_2_O_2_ on hBMSC after 2 h, and after 7 days of treatment with control-DMEM and original MSC-CM after 2 h exposure to 500 μM H_2_O_2_ was analyzed by the FITC-Annexin V Apoptosis Detection Kit II (BD Biosciences, Franklin Lakes, NJ, USA) using flow cytometry, following the manufacturer’s instructions. Briefly, cells were washed with cold PBS, trypsinized, resuspended in a binding buffer solution, and then incubated with FITC-Annexin V and PI solution for 15 min at room temperature in the dark. Then, a binding buffer was added, and cells were analyzed with BD Accuri C6 Cell Analyzer (BD Biosciences, Franklin Lakes, NJ, USA). Final data analysis was performed using flow cytometry data analysis software (FlowJo V10).

### 4.8. Intracellular ROS Assay

ROS generation in hBMSC exposed to 500 μM H_2_O_2_ followed by control-DMEM and original MSC-CM for 7 days was measured using a fluorometric intracellular ROS assay (Sigma-Aldrich, St. Louis, MO, USA) according to the manufacturer instructions. Cells were incubated with ROS detection reagent under 5% CO_2_, 37 °C for 1 h. Fluorescence was then measured at 490 nm excitation, 525 nm emission using the microplate reader. ROS generation is presented as a percentage compared to the control-DMEM-treated group.

### 4.9. Superoxide Dismutase (SOD) Assay

The effect of treatment with control-DMEM and original MSC-CM, on the level of total intracellular SOD after exposure to 500 μM H_2_O_2_, was studied after 7 days using a colorimetric SOD assay kit (Sigma-Aldrich, St. Louis, MO, USA), following the manufacturer’s instructions. In brief, cells were washed twice with PBS before adding the RIPA buffer (Thermo Fisher Scientific, Rockford, IL, USA) to each well to lyse the cells on ice for 10 min. The recommended volume of cell lysate was incubated with water-soluble tetrazolium salt and SOD enzyme for 20 min at 37 °C. Absorbance was measured at 450 nm using the microplate reader. Enzyme activity was calculated as a percentage of inhibition rate based on the manufacturer’s instructions.

### 4.10. Osteogenic Differentiation

To study the effect of H_2_O_2_ on the osteoblastic differentiation potential of hBMSC, cells were exposed to increasing concentrations of H_2_O_2_ for 2 h, as mentioned above. For the induction of osteoblastic differentiation, cells were washed with PBS and cultured in an osteogenic medium (OM) growth medium supplemented with L-ascorbic acid β-glycerophosphate, and dexamethasone (all from Sigma-Aldrich, St. Louis, MO, USA). Media were changed twice a week. After 21 days, mineralization of the extracellular matrix (ECM) was assessed by staining with 2% Alizarin Red S solution (Sigma-Aldrich, St. Louis, MO, USA). Images were taken using the inverted microscope. For quantification, the stain was dissolved in 1% cetylpyridiniun chloride (Sigma-Aldrich, St. Louis, MO, USA) and absorbance was then measured at 540 nm using a microplate reader. To study the effect of original MSC-CM treatment on the osteogenic differentiation capacity of hBMSC after exposure to 500 µM H_2_O_2_ for 2 h, cells were cultured in three different culture conditions: OM, 500 μM H_2_O_2_ then OM (H_2_O_2_+OM), and 500 μM H_2_O_2_ then OM with original MSC-CM (H_2_O_2_+OM+MSC-CM). Media were changed twice a week. After 14 days, ALP activity was tested using a BCIP/NBT alkaline phosphatase color development kit following the manufacturer’s instructions (Sigma-Aldrich, St. Louis, MO, USA). After 21 days, mineralization was evaluated using Alizarin Red S staining and quantified as mentioned above.

### 4.11. Real-Time Quantitative Polymerase Chain Reaction (qPCR)

Total RNA was manually extracted from hBMSC exposed to 500 µM H_2_O_2_ for 2 h and treated with control-DMEM or original MSC-CM for 3 days to detect mRNA expression levels of pro-and anti-apoptotic related genes: caspase3 (CASP3), B-cell lymphoma 2 (BCL-2), and Bcl-2 Associated X-protein (BAX) (Supplementary Table S4). Using a similar method, total RNA was also extracted from hBMSC cultured in OM, H_2_O_2_+OM, and H_2_O_2_+OM+MSC-CM for 7 and 14 days to detect mRNA expression levels of osteogenic-related genes: runt-related transcription factor 2 (RUNX2), collagen type I (Col 1α2), bone morphogenic protein 2 (BMP-2), and osteopontin (SPP1) (Supplementary Table S4). The purity and amount of the extracted RNA were quantified using Nanodrop ND-1000 Spectrophotometer (Nanodrop Technologies, Wilmington, DE, USA). cDNA was then synthesized using a high-capacity cDNA Reverse Transcription kit (Applied Biosystems). Real-time qPCR was performed with the TaqMan Fast Universal PCR Master Mix (Applied Biosystems, Vilnius, Lithuania) using the StepOne™ Real-Time PCR System (Applied Biosystems, Singapore, Singapore). mRNA expression levels of the targeted genes were analyzed as relative expression to glyceraldehyde 3 phosphate dehydrogenase (GAPDH) (Supplementary Table S4) using the comparative Ct method.

### 4.12. Immunofluorescence Staining

Immunofluorescence staining was performed in hBMSC cultured in OM, H_2_O_2_+OM, and H_2_O_2_+OM+MSC-CM for 7 days to detect Col 1α2 secretion. Briefly, cells were fixed with 4% paraformaldehyde (Sigma-Aldrich, St. Louis, MO, USA) for 15 min at room temperature. Cells were permeabilized with 0.2% Triton X-100 (Sigma-Aldrich, St. Louis, MO, USA) for 10 min, and nonspecific binding sites were blocked using 10% normal goat serum (Dako, Glostrup, Denmark) in 1% BSA in PBS for 1 h. Cells were incubated with mouse polyclonal anti-Col 1α2 antibody (Abcam, Cambridge, UK, dilution 1:200) at 4 °C overnight. After several washes with PBS, cells were incubated with the secondary antibody goat anti-mouse Alexa Fluor 546 IgG (Abcam, Cambridge, UK, dilution 1:500) for 45 min in dark. Phalloidin Atto488 (Sigma-Aldrich, St. Louis, MO, USA, dilution 1:500) was added with the secondary antibody for staining of the actin cytoskeleton. After washing with PBS, nuclei were stained with 4’,6-diamidino-2-phenylindole (DAPI) (Sigma-Aldrich, St. Louis, MO, USA, dilution 1:2000) for 5 min in the dark. Fluorescence was visualized using an inverted fluorescence microscope (Eclipse Ti, Nikon, Tokyo, Japan).

### 4.13. Statistical Analysis

Each experiment was conducted with cells from three donors. Data were presented as an average of the donors and analyzed by Graph Pad Prism 9 software (GraphPad, San Diego, CA, USA) using Student’s t-test and one-way ANOVA to test the difference between the means of different treated groups. *p*-value < 0.05 was considered statistically significant.

## 5. Conclusions

In summary, we examined the antioxidant effects of MSC-CM on cultured hBMSC using an in vitro oxidative stress model. Our results demonstrated that MSC-CM possesses a group of proteins with antioxidant properties and has a protective effect against oxidative stress-induced damage on hBMSC. We believed that this effect might be due to the synergistic effect between the antioxidant proteins and other biological factors present in MSC-CM, including factors with anti-apoptotic, angiogenic, and osteogenic properties. Accordingly, the therapeutic approach using MSC-CM has the potential to improve bone repair in patients with bone diseases related to oxidative stress.

## Data Availability

Data can be available upon request from the corresponding author.

## Supplementary Materials

Supplementary Materials can be found at https://www.mdpi.com/article/10.3390/ijms222413458/s1.
