# Supplementary material for: Conditioned Medium from Bone Marrow Mesenchymal Stem Cells Restored Oxidative Stress-Related Impaired Osteogenic Differentiation
Source: Int J Mol Sci. 2021 Dec 15;22(24):13458. doi: 10.3390/ijms222413458 (PMC8706339; doi:10.3390/ijms222413458)
Supplement: Supplementary file 1 [file ijms-22-13458-s001.zip › ijms-1466722-supplementary.pdf]

Supplementary materials

Table S1. Enrichment of molecular function GO terms using DAVID. GO terms were ranked according to count (%). False discovery rate (FDR) corrected p-values were determined using the Benjamini-Hochberg procedure. Top 10 are depicted.

GO Term	Description	Count (%)	FDR
GO:0007165	Signal transduction	7.4	7.3e-1
GO:0098609	Cell-cell adhesion	6.8	6.1e-60
GO:0007155	Cell adhesion	6.1	1.5e-19
GO:0006508	Proteolysis	4.9	2.4e-6
GO:0055114	Oxidation-reduction process	4.9	3.6e-3
GO:0030198	Extracellular matrix organization	4.3	8.6e-31
GO:0016032	Viral process	4.1	8.3e-14
GO:0000398	mRNA splicing, via spliceosome	3.9	2.6e-20
GO:0043066	Negative regulation of apoptotic process	3.9	3.8e-3
GO:0008284	Positive regulation of cell proliferation	3.9	7.8e-3

Table S2. Enrichment of molecular function GO terms using STRINGdb. False discovery rate (FDR) corrected p-values were determined using the Benjamini-Hochberg procedure. GO terms were ranked according to strength. Top 10 are depicted.

GO Term	Description	Count	Strength	FDR
GO:0051920	Peroxiredoxin activity	7 of 7	1.02	3.1e-3
GO:0044388	Small protein activating enzyme binding	5 of 5	1.02	2.7e-2
GO:0008379	Thioredoxin peroxidase activity	5 of 5	1.02	2.7e-2
GO:0048407	Platelet-derived growth factor binding	9 of 11	0,93	9.1e-4
GO:0030020	Extracellular matrix structural constituent conferring tensile strength	21 of 28	0,9	2.1e-8
GO:0004298	Threonine-type endopeptidase activity	16 of 21	0,9	1.8e-6
GO:0032050	Clathrin heavy chain binding	6 of 8	0,9	2.3e-2
GO:0031995	Insulin-like growth factor ii binding	6 of 8	0,9	2.3e-2
GO:0015037	Peptide disulfide oxidoreductase activity	9 of 13	0,86	2.3e-2
GO:0043394	Proteoglycan binding	25 of 37	0,85	2.0e-9

Table S3. Information concerning antioxidant proteins identified in AOD.

Accession	Gene ID	Protein Name	Function
**Q06830**	PRDX1	Peroxiredoxin-1	Catalyzes the reduction of H_2O_2 and organic hydroperoxides to water and alcohols. Protects cells against oxidative stress.
**P32119**	PRDX2	Peroxiredoxin-2	Catalyzes the reduction of H_2O_2 and organic hydroperoxides to water and alcohols. Protects cells against oxidative stress.
**P30048**	PRDX3	Thioredoxin-dependent peroxide reductase	Catalyzes the reduction of H_2O_2 and organic hydroperoxides to water and alcohols. Protects cells against oxidative stress.
**Q13162**	PRDX4	Peroxiredoxin-4	Catalyzes the reduction of H_2O_2 and organic hydroperoxides to water and alcohols. Protects cells against oxidative stress.
**P30044**	PRDX5	Peroxiredoxin-5	Catalyzes the reduction of H_2O_2 and organic hydroperoxides to water and alcohols. Protects cells against oxidative stress.
**P30041**	PRDX6	Peroxiredoxin-6	Catalyzes the reduction of H_2O_2 and organic hydroperoxides to water and alcohols. Protects cells against oxidative stress.
**P00441**	SOD1	Superoxide dismutase	Attaches to Cu and Zn to break down superoxide radicals. Protects the cell from reactive oxygen species toxicity.
**P99999**	CYCS	Cytochrome c	Transfers electrons to the cytochrome oxidase complex. Involved in apoptosis.

Table S4. Gene primers (all from Applied Biosystems)

Gene Type	Gene	Assay ID
**Reference**	GAPDH	Hs02758991_g1
**Apoptotic**	CASP3	Hs00234387_m1
**Pro-apoptotic**	BAX	Hs00180269_m1
**Anti-apoptotic**	BCL-2	Hs00153350_m1
**Osteogenic**	Col1a2 (Collagen type I)	Hs01028956_m1
**Osteogenic**	BMP-2 (bone morphogenetic protein 2)	Hs00154192_m1
**Osteogenic**	SPP1 (Osteopontin)	Hs00960942_m1
**Osteogenic**	RUNX2 (runt-related transcription factor 2)	Hs01047973_m1

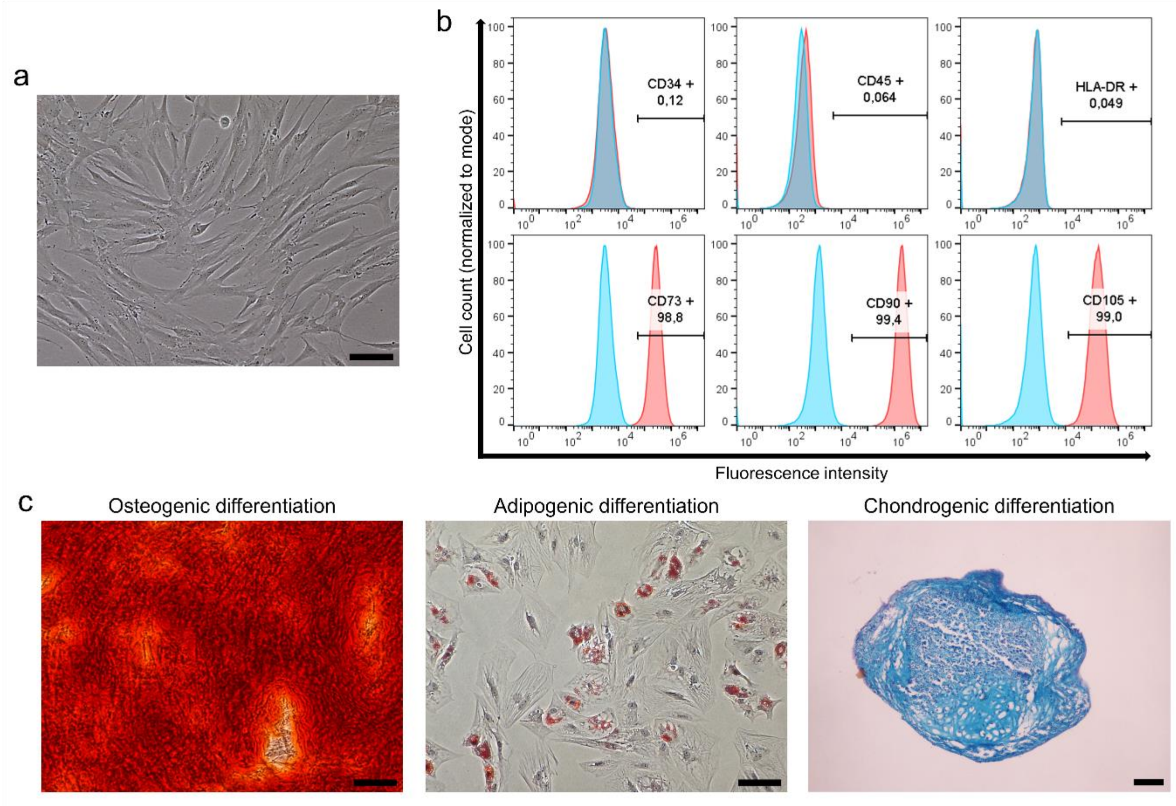

Figure S1. Characterization and multi-differentiation of hBMSC. (a) Microscopic image showing fibroblastic morphology of hBMSC. Scale bar 100 µm. **(b)** Histograms showing percentage of cell surface marker expression using flow cytometry analysis of hBMSC, antibody control (blue) and the stained cells (red). **(c)** Images showing tri-lineage differentiation into osteogenic, adipogenic and chondrogenic lineages confirmed by Alizarin red, Oil red O and Alcian blue staining, respectively. Scale bars 100 µm.
